# In Memoriam

**Published:** 1885-07-20

**Authors:** 


### IN MEMORIAM.

Death is a sad event at all times, and under all circumstances, but when it comes and suddenly cuts down one whose dawning manhood is prophetic of most fair fulfilment we are so awed, if not bewildered, by a Providence like this we can only take refuge in silence, and the comforting thought,

"Thou doest all things well!"

While we feel that, as our short-sighted vision is unable to pierce the veil of futurity, we know not from what evil to come, a loving Heavenly Father has taken His child to Himself!

Dr. T. S. FEW was born February 21, 1864, graduated with the highest honor at the Southern Medical College of Atlanta, Ga., in March, 1885. He immediately returned to his home in South Carolina, and soon was attacked with typhoid phneumonia and died, after a few days' illness, just as he had manfully girded himself for an honorable warfare in life—to become a blessing to his family and to suffering humanity, and to honor the profession of Medicine, in which he had so faithfully fitted himself to do a noble work.

As a student in the Southern Medical College ,our young professional brother had endeared himself to his classmates by his gentle disposition and genial companionship, and he was beloved by the entire Faculty for his studious application, his affectionate and respectful obediance, and the faithful discharge of his whole duty. Above all, he was conspicuous in his conduct for those elevating principles that never fail to distinguish the true Christian gentleman wherever he may be, and to win the affection and respect of all with whom he came in contact.

Though Dr. Few graduated at our Institution with noteworthy distinction, and received the medal for the greatest proficiency in all the branches of Medicine, and thus was prepared to win an enviable position in his chosen profession, yet, we learn, that when Death came so unexpectedly to summon him from all this earthly promise of honor and usefulness, he did not utter one regret or one rebellious word. "But," as his almost heartbroken mother wrote me, "when informed by his attending physician that he must die, he merely said, 'Where is my mother; does she know it?' On being told that she did, and was then praying for him, he called for her and asked her to kneel by his bedside and pray. Then turning to his father and brother—the only other members of the family—he said, ' Meet me in Heaven ! ' Afterwards, with his feeble voice, he tried to sing his favorite hymn, ' Pass me not, O gentle Savior,' and thus himself passed away to be forever with the Savior he so loved on earth." Dr. Few united with the Methodist Church when seventeen years of age, and, as those who knew him testify, was an upright and consistent Christian to the day of his death.

With his mother's letter, I also received one from his medical preceptor, Dr. S. B. Crawley, of O'Neal, S. C., and would be glad if space permitted to copy it entire. I was struck with such remarks as these in the letter : " As a member of society, Dr. Few's character was without blemish. . . He was under my instruction one year before entering College, and during the whole time he never once faltered or evaded duty." And again : " One of his prominent characteristics should be emphasized : he was always ready and willing to heed the judicious councils of his elders, or recognized superiors, and to give preference to their views and their judgment." These statements coincide with the words of Dr. Few's mother, when she says in her letter to me: " Sidney, from infancy, was always kind and affectionate to all, and especially obedient to his parents, while he was devoted to his mother. When at College he wrote to me every week, and thanked me again and again for my loving interest in his welfare and for my maternal advice, by which he always tried to profit."

In these days of self-assertion and assumption of superior wisdom in so many of our young people, and their rebellion against the advice of elders and parents, such praise as the above is the highest eulogy that can be given a young man, whether his position in life be humble or exalted.

And I do not know that the removal by death of such a son is an occasion for most sorrow or joy. We know he is not lost, but only gone before, and we also know his beautiful short life is not lost to the world, for to us who remain behind it is left as a shining example to be loved and emulated ; and while his absence from the body may cause the hearts of those who loved him most on earth to greatly mourn, I feel assured there were joyful shouts in Heaven when the glorified spirit swept triumphantly through the swinging portals, and heard the welcome plaudit, " Well done, good and faithful servant, enter thou into the joy of the Lord."

May God comfort the sorrowing mother and friends of this dear young brother, and enable them, and all his classmates and medical instructors, to meet him in the beautiful Hereafter of God and the soul ! His noble life, short as it was, and his happy, peaceful death should convince every member of the profession he so loved that to be a Christian physician in its best and truest significance is the most exalted position to which we of the brotherhood could aspire.

